# Physical and mental health outcomes of an integrated cognitive behavioural and weight management therapy for people with an eating disorder characterized by binge eating and a high body mass index: a randomized controlled trial

**DOI:** 10.1186/s12888-022-04005-y

**Published:** 2022-05-24

**Authors:** Phillipa Hay, Marly Amorim Palavras, Felipe Quinto da Luz, Sérgio dos Anjos Garnes, Amanda Sainsbury, Stephen Touyz, José Carlos Appolinario, Angélica Medeiros Claudino

**Affiliations:** 1 School of Medicine, Translational Health Research Institute, Western Sydney University, 1797 Locked Bag Avenue, Sydney, 2751 Australia; 2 Eating Disorders Program (PROATA), Department of Psychiatry, Universidade Federal de São Paulo (UNIFESP), Rua Major Maragliano 241, São Paulo, SP 04017-030 Brazil; 3 Eating Disorders Program (AMBULIM), Faculty of Medicine, Universidade de São Paulo (USP), Rua Dr. Ovídio Pires de Campos, 785, São Paulo, SP 05403-010 Brazil; 4 Boden Collaboration for Obesity, Nutrition, Exercise and Eating Disorders, Faculty of Medicine and Health, Charles Perkins Centre, The University of Sydney, Sydney, 2006 Australia; 5 School of Human Sciences, The University of Western Australia, Perth, 6009 Australia; 6 Inside Out Institute and School of Psychology, Charles Perkins Centre, SLHD and The University of Sydney, Sydney, 2006 Australia; 7 Obesity and Eating Disorders Group – Institute of Psychiatry, Federal University of Rio de Janeiro (UFRJ), Avenida Pedro Calmon 550, Rio de Janeiro, RJ 21941-901 Brazil

**Keywords:** Binge-eating disorder, Bulimia nervosa, Therapy, Weight loss, Quality of life

## Abstract

**Background:**

Bulimia nervosa (BN) and binge eating disorder (BED) are eating disorders (EDs) characterized by recurrent binge eating. They are associated with medical complications, impaired adaptive function and often a high BMI, for which a multidisciplinary treatment approach may be needed. This study explored the efficacy of a novel intervention integrating Cognitive Behavioural Therapy- Enhanced (CBT-E) and weight management for people with recurrent binge eating episodes and high BMI with respect to physical, psychopathological and quality of life outcomes.

**Methods:**

Ninety-eight adults diagnosed with BN, BED, or Other Specified/Unspecified Feeding or Eating Disorder (OSFED/UFED) and BMI ≥ 27 to <40 kg/m^2^ were randomized to a multidisciplinary approach, the Healthy APproach to weIght management and Food in Eating Disorders (HAPIFED) or to CBT-E. Metabolic parameters, health-related quality of life, general psychological and ED symptoms and ED diagnostic status outcomes are reported. Data were analyzed with mixed effects models adopting multiple imputed datasets where data were missing.

**Results:**

Both HAPIFED and CBT-E showed statistical significance for the time effect, with reduction in stress (*p* < 0.001), improvement in mental health-related quality of life (*p* = 0.032), reduction in binge eating severity (*p* < 0.001), and also in global ED symptoms scores (*p* < 0.001), with the significant changes found at end of treatment and sustained at 12-month follow-up. However, no statistical significance was found for differences between the interventions in any of the outcomes measured. Despite a high BMI, most participants (> 75%) had blood test results for glucose, insulin, triglycerides and cholesterol within the normal range, and 52% were within the normal range for the physical component of quality of life at baseline with no change during the trial period.

**Conclusion:**

Integrating weight and ED management resulted in comparable outcomes to ED therapy alone. Although adding weight management to an ED intervention had no adverse effects on psychological outcomes, it also had no beneficial effect on metabolic outcomes. Therefore, more intense weight management strategies may be required where indicated to improve metabolic outcomes. Safety will need to be concurrently investigated.

**Trial registration:**

US National Institutes of Health clinical trial registration number NCT02464345, date of registration 08/06/2015. Changes to the present paper from the published protocol paper (Trials 18:578, 2015) and as reported in the Trial registration (clinicaltrials.gov) are reported in Supplementary File 1.

**Supplementary Information:**

The online version contains supplementary material available at 10.1186/s12888-022-04005-y.

## Background

Eating disorders (EDs) are characterized by food, weight and body shape concerns, as well as by behaviours such as recurrent binge eating, inappropriate compensatory weight control mechanisms and dietary restriction, impaired physical and mental health-related quality of life, and poor psychosocial functioning [1]. Bulimia nervosa (BN) and binge eating disorder (BED) are the most prevalent diagnoses among the main ED categories. Both are distinguished by the presence of recurrent binge eating episodes associated with a sense of loss of control. The global lifetime prevalence estimates for BN is 1.9% in women and 0.6% in men, and for BED, 2.8% in women and 1.0% in men and there has been a doubling of the global prevalence of EDs from 3.5 to 7.8% between 2000–2006 and 2013–2018 [2]. When all categories of ED are considered, the most prevalent diagnosis is Other Specified Feeding or Eating Disorder (OSFED) with a lifetime prevalence weighted mean of 4.3% for women and 3.6% for men [2]. Thus overall the most common EDs are BED and OSFED-type BN and BED of low frequency and/or limited duration which together account for approximately half of the high number of disability adjusted life years (DALYs) that are due to EDs worldwide (6.6 million DALYs) [3]. Prevalence rates of ED behaviours are also increasing. An Australian general population study showed a significant increase in the prevalence of binge eating (3.2 to 11.1%) and binge eating associated with high body mass index (BMI) (1.0 to 5.7%) between 1995 and 2015 [4]. A longitudinal study from the USA found a high rate (25.3%) of disordered eating behaviours (binge eating or unhealthy weight control) in young adults with a high BMI when compared to underweight, normal or overweight status [5].

Individuals with recurrent binge eating episodes show impairment in physical health-related quality of life due to gastrointestinal, cardiac, endocrine and other medical complications [6, 7]. For BN, the most common complications are consequences of recurrent use of purgative compensatory behaviours (self-induced vomiting, and abuse of laxative and diuretics) [6]. In individuals with BED, an association with high weight contributes to metabolic (including hepatic) and musculoskeletal disorders [8]. In a USA general population survey, over 90% of people with BN or BED were found to have a lifetime psychiatric disorder (depression, anxiety, posttraumatic stress, substance use, and/or personality disorder) [9]. When compared to healthy populations, individuals with ED have significantly poorer health-related quality of life, more frequent hospitalizations or emergency department visits and higher outpatient care, despite low use of ED specific treatment. A systematic review highlighted that the economic burden is also high (e.g., annual healthcare costs for BN and BED varies from €888 to €18,823). The authors argued that obesity may be an important feature contributing to these costs [10].

Several evidence-based clinical guidelines recommend specific psychological interventions for individuals with recurrent binge eating episodes [11]. Guerdjikova et al. (2019) [12] endorse a multidisciplinary team involving a psychologist, a dietician, and a psychiatrist for supporting individuals with recurrent binge eating episodes, mainly when associated with high BMI. In this context and with calls for more research [13], HAPIFED (a Healthy APproach to weIght management and Food in Eating Disorders) was developed. It is a novel manualized approach integrating Cognitive Behavioural Therapy-Enhanced (CBT-E) with Behavioural Weight Loss Therapy (BWLT) for individuals with recurrent binge eating episodes comorbid with high BMI [14].

We have conducted a single-blind randomized controlled trial (RCT) testing the efficacy of group-based HAPIFED compared to group-based CBT-E for participants with BN, BED, OSFED (type BN or BED), or unspecified feeding or eating disorders (UFED), with a BMI ≥ 27 and < 40 kg/m^2^. The main outcome was maintained weight loss at 12-month follow-up. At 12-month follow-up both interventions had just over 20% of participants achieving ≥5% reduction in body weight, and a significant time effect was found for 5% body weight loss from baseline to end of treatment that was sustained at the 12-month follow-up. There were no significant differences between groups in the primary outcome (weight loss) or secondary outcomes related to ED symptoms. However, HAPIFED was favoured over CBT-E for the reduction in purging behaviour (x^2^(3) = 10.35, *p* = 0.016), as well as for the improvement in binge remission rates (i.e., a greater number of individuals who were abstinent from binge eating in the last 3 months), specifically at the 12-month follow-up (x^2^ (1) = 3.97, *p* = 0.049) [15]. The purpose of this current study was to report the results of the other physical and psychopathological secondary outcomes from this trial, namely indicators of metabolic health, physical and mental health-related quality of life, general psychopathology, severity of binge eating, global self-reported ED symptoms and remission of ED diagnoses. We hypothesized that, at the end of the trial, participants who received HAPIFED would have better indicators of metabolic health and physical health-related quality of life and similar improvements in ED and general psychiatric symptoms and in mental health-related quality of life compared to those receiving CBT-E intervention.

## Methods

### Design

This single blind, two-arm RCT, was conducted at the Eating Disorders Program (PROATA)/Department of Psychiatry of the Universidade Federal de São Paulo (UNIFESP), Brazil. Methods are also reported in Palavras et al. (2021; 2015) [15, 16] and changes from the published protocol paper [16] and as reported in the Trial registration (clinicaltrials.gov) are reported in the Supplementary online File 1.

### Participants and procedures

Participants were recruited and enrolled from July 2015 to November 2017, with recruitment through the waiting list of an outpatient university program specialized on the treatment of EDs (PROATA/UNIFESP) and via oral and written media advertisements, comprising both clinical and community recruitment. A brief telephone or email screening checked eligibility of 589 respondents to this recruitment. The inclusion criteria were age ≥ 18 years, any sex, presence of recurrent binge eating with diagnosis of BN, BED, or OSFED/UFED according to the criteria of the Diagnostic and Statistical Manual of Mental Disorders, fifth edition (DSM-5) [1] and/or the International Classification of Diseases, eleventh version (ICD-11; note at the time of the study these were proposed criteria) [17], and a BMI ≥ 27 kg/m^2^ and < 40 kg/m^2^. Exclusion criteria were presence of a current diagnosis of bipolar disorder, psychosis and/or a high level of suicide risk, use of weight loss medication, history of bariatric surgery, a clinical condition interfering with appetite regulation (e.g., Prader-Willi or Cushing syndromes) and engagement in current psychological therapy for ED.

Participants who were eligible were invited to attend a first in-person interview at our research facility. At this first in-person interview, the study was explained in depth, eligibility criteria were re-checked, and those who met the eligibility criteria completed an informed consent form. In sequence, socio-demographic status, anthropometric measures, medical history, psychiatric comorbidities, and self-report questionnaires (evaluating ED symptoms, depression, anxiety, stress symptoms and health-related quality of life) were collected. The participants had a fasting blood test. This was collected by Associação Fundo de Incentivo à Pesquisa, a non-profit private institution that works with diagnostic medicine and research, previously chosen for this RCT and in partnership with UNIFESP. Participants approved in the first interview were invited to a second in-person semi-structured interview to confirm ED diagnoses and to collect more specific information about the ED symptomatology.

### Sample size

An estimate of a moderate effect size (i.e., 0.6) between groups was applied for the sample size calculation, considering weight loss as the primary outcome of the RCT study. According to Cohen’s tables, a minimum of 72 participants in total (36 per group) were required based on a power of 0.8 and alpha = 0.05. Considering attrition, the sample size was estimated to be 100 in total (50 per treatment arm).

### Randomization and treatment fidelity

Eligible participants were randomized into blocks of 20 (1:1 ratio) for both groups, being allocated in 10 groups with 10 participants in each intervention. Five groups received the experimental intervention (HAPIFED) and five groups received the control intervention (CBT-E). This allocation of intervention to the 10 groups was conducted by an investigator (PH) external to the site, using a computer-generated sequence facilitated by the sealed envelope website (www.sealedenvelope.com). Only three researchers (PH, AMC and MAP) and the therapists had access to this allocation. The randomization of the 98 participants occurred after the first individual session in the interventions, which was the same for all participants regardless of intervention (described below), and they were blind to their group allocation until completion of the RCT (end of 12-month follow-up). The statistician and the recruiters (with the exception of the dietitian who performed anthropometric measurements) were also blind to treatment group.

Four therapists were trained in both interventions. To minimize non-specific therapists’ effect, two therapists conducted HAPIFED and two different therapists conducted CBT-E. When two new groups started, the therapists swapped so that the first two conducted CBT-E, and the other two conducted HAPIFED, and so forth. Experts (PH, AS and Jessica Swinbourne) in HAPIFED and CBT-E provided monthly telephone facilitated supervision to the Brazilian team. Furthermore, a senior researcher (PH) conducted face-to-face supervision during twice yearly visits to Brazil during the three years in which the RCT was conducted. During the RCT, one author (FQdL) checked a random sample (10%) of de-identified digitally recorded audiotaped sessions to monitor fidelity to the manual and advised if there was therapist drift.

### Description of intervention

HAPIFED (experimental intervention) and CBT-E (control intervention) were equally delivered over 30 sessions. The first session (session 1) for both HAPIFED and CBT-E was an individual session where the ED history was asked, a personalized formulation derived, and points related to the treatment clarified. The 29 subsequent sessions were offered in a group format, comprising of sessions twice weekly for the first 4 weeks, and then weekly until the end of treatment, over a total time of 6 months. Descriptions of the topics of each of the 29 group sessions for the HAPIFED and CBT-E are provided in Supplementary File 2, and similarities and differences between the therapies, as implemented in this study, are summarized in Supplementary File 3. Each participant received a workbook in both interventions. One of the handouts is provided with a table to enter their measured weight and in which session if they choose (i.e. it was not mandatory). The weight measures are neither plotted on a graph, nor discussed during the sessions.

HAPIFED is a manualized program composed of five stages [14]. *Stage one* (sessions 2–11): mainly offers psychoeducation about ED symptoms and behaviours and weight, in relation to mental and physical risks. Real-time self-monitoring is introduced and encouraged, with the additional evaluation of internal cues related to hunger and satisfaction. *Stage two* (session 12): as in CBT-E, the personal formulation process is revised and improved. Here a joint review of progress/identifying barriers to change is conducted and each participant had the opportunity to discuss/share/reflect on her/his own formulation. In other sessions, when issues emerged for the participant relevant to the formulation, the therapists explored these, linking theory to practice, with reference to the situation referred by the participant. *Stage three* (sessions 13–19): the focus is on behavioural change and monitoring through the practice of specific behavioural skills, e.g. activities that emphasize the identification of body’s positive aspects, tasks that promote better self-care, and skills training in progressive muscular behavioural relaxation, etc. *Stage four* (sessions 20–27): highlights the relevance of changing unhealthy beliefs and attitudes for modification of unsuitable ED behaviours. *Stage five* (sessions 28–30): follows the same orientation offered by CBT-E for the management of relapses and recheck of healthy cognitive and behavioural strategies. Features unique to HAPIFED (not found in CBT-E) include psychoeducation around ED and high weight, the monitoring of internal cues of hunger and satiety, dietitian-led nutritional counselling, and weight loss strategies including increased physical activity. Also, two home visits by an occupational therapist for evaluation of the domestic environment and the person’s daily routines, and advice on improvements for food preparation and physical activities (in stages 1 and 3) were included in HAPIFED and not in CBT-E. The HAPIFED manual is available in da Luz et al., 2017 [14].

The control intervention was the CBT-E treatment manual [18], an enhanced version of CBT, considered to have the best evidence-base as therapy for BED and BN [19–21], including in group format [22]. An early meta-analysis of RCTs of group therapies for BN found evidence for the efficacy of group therapy when compared with no treatment (albeit rated low-quality evidence), and insufficient evidence when compared with individual therapy [22]. However, more recently, Wade et al.’s [23] RCT of an ED transdiagnostic sample (57.5% with BN and 5% with BED), showed CBT-E delivered in a group format was effective, feasible and acceptable when compared with a wait-list control condition. A recent meta-regression analysis found the group format to be superior to individual therapy for BED [21]. CBT-E is available in both a focused and broad version. The focused version deals specifically with the ED psychopathology and it is delivered in 20 sessions for individuals who do not have a body weight in the underweight range. In a complementary way, the broad version adds three modules addressing external mechanisms – perfectionism, low self-esteem and interpersonal difficulties – that can contribute to the ED severity, and the manual advises that these can be used to prolong the number of sessions (e.g., up to 30 sessions) [18]. The group format and the broad version of CBT-E (with 30 sessions to match HAPIFED in number of sessions) were selected for this trial. This trial adapted group format CBT-E to 30 sessions, with all the 20 sessions of the original version (for which there is evidence) included, and some of them extended in booster sessions, where topics could be more deeply explored and discussed. The guide advises that these modules should only be used when one or more of these external mechanisms are determined to be an obstacle to progress at the Stage 2 review. However, we considered that the inclusion of all additional modules was very suited in a group setting where it was likely they would be applicable for at least one or more participants, and the group setting facilitates vicarious learning and support by participants. For example, group members who have higher levels of self-esteem were able to share how their self-esteem was enhanced through previous life or therapeutic experiences. Thus, we anticipated that our extended version in this group delivery may have added efficacy to the original 20 session version, although this comparison has not been directly made in this trial. CBT-E is delivered in four stages. *Stage one* (sessions 2–7): highlights the participant engagement, presents the real-time self-monitoring and offers psychoeducational material on the potential benefits of regular eating and in-session weighing. *Stage two* (sessions 8–9): assesses progress, reviews the formulation, and discusses any barriers related to the treatment. *Stage three* (sessions 10–17)*:* discusses maintaining mechanisms of the ED symptomatology (e.g., mood intolerance). *Stage four* (sessions 18–20): prepares for the end of the treatment and continued progress [18]. Although evidence of CBT-E mainly comes from the focused version individually applied, and the evidence that supports group treatment against no treatment or waitlist control groups is still weak, the existing evidence supports further testing of group CBT-E, especially in face of costs and availability of individual treatment worldwide and the need to provide treatment for more patients at the same time.

Following the 30 sessions, both groups received four additional follow-up sessions over the 6 months after the end of the active intervention. The same therapists (who delivered the active intervention) conducted these four additional sessions, which aimed to address lapses, encourage progress, and give support for continued improvement.

It should be noted that in the original version of CBT-E broad version, mood intolerance was a fourth additional module and has since been incorporated in the focused form. Thus, CBT-E broad version has three additional modules– clinical perfectionism, low self-esteem and interpersonal problems – which in this study were sessions 21–26 of CBT-E. HAPIFED includes the mandatory additional module addressed mood intolerance and also has a session addressing interpersonal problems. Thus, in this study, HAPIFED included all of CBT-E focused content, as well as, issues to do with weight control and interpersonal function, but not the remaining two broad CBT-E modules (clinical perfectionism and low-self-esteem), while CBT-E included the focused version and all the three broad modules, but nothing specifically addressing weight control/weight loss. We chose not to include all additional modules of CBT-E in HAPIFED. As the purpose was to also aid weight loss management, HAPIFED included four sessions delivered by nutritionist that offered psychoeducation about e.g., the Famine Reaction/Why diets fail? (session 3), regular eating (session 4), healthy and unhealthy exercise (session 5 – here also facilitated with the occupational therapist) and healthy eating for weight loss (session 7). However, since participants were included with a diagnosis of BN which is associated with high weight and body shape concerns, and dietary restriction, the research team took special care when discussing these topics during the sessions, to ensure that there was no worsening of ED symptoms. In sum, we chose to include all three additional modules in CBT-E as we were extending it from 20 to 30 sessions, and it provided additional content.

For the purpose of group delivery, no changes were made to manual content. Group boundaries and confidentiality rules were set up in the first group session i.e., "what is said in the group stays in the group". Individual weighing was completed in a private room by the therapist prior to each group session. Records and homework exercises were presented and discussed in the group setting, but not all participants were required to share their records/homework at every session. People benefitted from the vicarious learning and support of other group members. Meeting outside the group was discouraged. A group “WhatsApp” was created by the therapists for relevant communications, e.g., absences due to public transport strikes, and the therapists’ mobile was also available for information the person preferred to keep confidential.

### Assessments

All participants (whether or not they completed the active intervention) were asked to be assessed at five time-points: baseline, middle of treatment (3 months), end of treatment (6 months), 6-month follow-up (6 months after end of treatment and 12 months from baseline) and 12-month follow-up (12 months after end of treatment and 18 months from baseline). All the instruments used in this RCT have been detailed in the previously published papers [15, 16], with all presenting suitable psychometric properties. Assessment comprised the following measures.

1. Socio-demographic (age, sex, race, occupation, marital status, education) and clinical (illness duration) variables were evaluated through a self-report questionnaire at baseline only.
2. Anthropometry namely waist and hip circumferences (mean of three measures), pulse and sitting blood pressure measured at baseline, end of treatment, 6 and 12-month follow-up. For the calculation of BMI (kg/m^2^), weight was measured using a calibrated scale at all five time-points. Height was measured using a stadiometer at baseline only.
3. Blood tests for measurement of fasting circulating concentrations of glucose, lipids (triglycerides, and HDL and LDL cholesterol) and insulin were collected at baseline and at the end of treatment.
4. General psychiatric symptomatology were assessed using the Mini International Neuropsychiatric Interview (MINI) [24] at baseline only.
5. ED diagnoses and symptomatology were assessed by expert psychologists and psychiatrists trained in the application of the Eating Disorder Examination (EDE) [25] at baseline, end of treatment, 6 and 12-month follow-up, considering both the DSM-5 [1] and ICD-11 [26] criteria. Cronbach’s α for the global EDE score in this study was 0.77 (22 items).
6. The following validated self-report questionnaires assessed general and ED symptoms and health-related quality of life at all time-points:
  6.1. Eating Disorder Examination Questionnaire (EDE-Q) [27] – with dietary restraint, eating, weight and shape concern subscales. Cronbach’s α for the global score was 0.81 (29 items).
  6.2. Binge Eating Scale (BES) [28] – for the presence and severity of binge eating behaviour. A cut-off of 17 has been determined as a clinically significant severity measure for Brazilian people with obesity seeking treatment for weight loss [29]. In this study Cronbach’s α was 0.84 (16 items).
  6.3. Depression, anxiety and stress scale – short form 21 items (DASS-21) [30, 31] – to assess the presence and severity of symptoms of depression, anxiety and stress. Scores ≤ 9 for depression, ≤ 7 for anxiety and ≤ 14 for stress are defined to be within a normal range. A Brazilian/Portuguese version has been validated [32]. In this study Cronbach’s α for depression, anxiety and stress were 0.89, 0.80 and 0.85, respectively (7 items for each subscale).
  6.4. The 12-item Short Form Health Survey (SF-12) v.1 [33] – assesses health-related quality of life in physical and mental health domains. The mean scores considered ‘normal’ are 50 points for both components in a Brazilian population [34]. In this study Cronbach’s α was 0.77 for the 6 physical component summary items, and 0.78 for the 7 mental component summary items.

### Statistical analyses

Clinical and demographic data, as well as outcome variables, were analysed using baseline univariate between-group tests. Two independent sample *t* tests were used to compare the means of outcomes with normal distribution considering the control group and the experimental group. For categorical variables, the Fisher’s exact test was used also considering treatment versus control groups. Data were inspected for normality and descriptive data were presented as mean (SD) and n (%) as appropriate.

The data were analyzed following the principle of intention to treat (ITT). For this purpose, missing data were imputed based on the multivariate and multinomial normal distribution using the Markov chain Monte Carlo (MCMC) algorithm [35], with 10 replications being performed. The results of the 10 replications were combined using Rubin’s rule [36, 37]. Continuous outcome variables were assessed using Generalized Estimation Equation (GEE) models with normal distribution and identity link function [38]. The GEE approach allows the incorporation of the dependence between the observations of the same individual resulting from the repeated measures carried out over time. Although this model assumes a normal marginal distribution, the GEE allows the assumption of normality to be relaxed in the distribution of dependent variables [38]. For evaluation of categorical (binary) outcome variables (i.e., whether or not a participant met the threshold criteria for a particular diagnosis), the GEE model was used again as a logit link function and binomial marginal distribution. In verifying the time effect, the five time points (baseline, middle of treatment, end of treatment and 6 and 12-follow-up), or in the case of metabolic health (blood markers and waist and hip circumferences) with only two available time points (baseline and end of treatment), were considered using pairwise comparison with Bonferroni correction. Cohen’s *d* was used to estimate effect sizes for continuous variables between the treatment and control groups. Small, moderate and large effect sizes were defined as 0.20–0.49, 0.50–0.79 and 0.80–1.00, respectively [39]. Statistical analyses were performed using the statistical software STATA 15 [40], and, for a small number of analyses (i.e., for baseline socio-demographic and clinical features) the SPSS version 20 for Windows [41].

### Outcomes

The following continuous outcome variables were entered in the analyses: (a) indicators of metabolic health (waist and hip circumferences and blood markers); (b) physical and mental health-related quality of life; (c) levels of depression, anxiety and stress; (d) severity of binge eating and global ED symptoms; and, (e) remission of ED diagnosis. The only categorical outcome variable in this paper was whether or not a participant met the threshold criteria for a particular diagnosis (i.e., one of BED, BN, or OSFED/UFED).

## Results

**Fig. 1:**
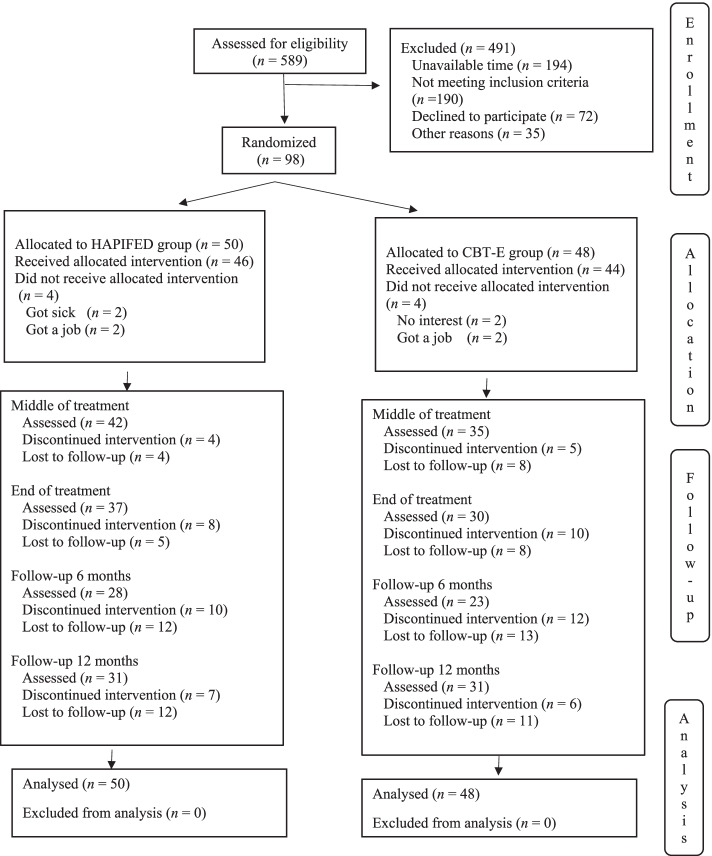
Participant flow chart. Footnote: Reprinted by permission from Springer Nature Customer Service Centre GmbH, first published in Eat Weight Disorders – Studies on Anorexia, Bulimia and Obesity with the title “Integrated weight loss and cognitive behavioural therapy (CBT) for the treatment of recurrent binge eating and high body mass index: a randomized controlled trial”, authors: Palavras MA, Hay P, Mannan H, da Luz FQ, Sainsbury A, Touyz S, Claudino AM. Copyright, 2021. 10.1007/s40519-020-00846-2

From 98 participants, 50 were randomized to HAPIFED (the experimental intervention) and 48 to CBT-E (the control intervention), with all being included in the ITT statistical analysis. The participant flow is shown on Fig. 1.

The *a priori* estimate was to include 100 participants, but difficulties with the recruitment and selection process took longer than expected, and only 98 participants could be included. Along the study time points, research attrition rates (number of participants who did not complete assessments) for the total sample (N = 98) were: 31.6% at the end of treatment; 48.0% at 6-month follow-up; and 36.7% at 12-month follow-up. Along the stages of the trial, no difference was observed for dropout between intervention groups. The final sample comprised 96% women, 75% white, 45% married, 43% tertiary educated, and 60% employed individuals, with a mean age of 40.55 (SD 11.70) years and a mean BMI of 33.68 (SD 3.31) kg/m^2^. Groups did not differ at baseline concerning these demographic and other clinical features (see Table 1).

**Table 1:** Baseline features of treatment groups (*n* = 98)

Feature	HAPIFED (***n =*** 50) Mean (SD)	CBT-E (***n*** = 48) Mean (SD)	***p*** ^a^
Weight, kg	88.96 (11.93)	89.59 (13.34)	0.805^a^
Body mass index, kg/m^2^	33.62 (3.19)	33.74 (3.47)	0.854^a^
Waist circumference, cm	105.18 (10.86)	104.9 (10.74)	0.901^a^
Hip circumference, cm	118.83 (8.29)	118.84 (8.57)	0.991^a^
Serum glucose, mg/dL	93.51 (8.08)	94.49 (10.97)	0.627^a^
Serum insulin, ulU/mL	13.57 (6.55)	12.64 (9.34)	0.583^a^
Serum triglycerides, mg/dL	147.66 (159.54)	123.98 (77.21)	0.371^a^
Serum HDL cholesterol, mg/dL	54.55 (11.35)	53.84 (12.04)	0.772^a^
Serum LDL cholesterol, mg/dL	117.50 (31.93)	113.84 (22.46)	0.529^a^
SF12 Physical health component summary	47.22 (10.49)	49.97 (9.08)	0.167^a^
SF12 Mental health component summary	34.31 (10.03)	36.92 (11.73)	0.238^a^
DASS – depression subscale	16.38 (10.86)	16.25 (11.70)	0.955^a^
DASS – anxiety subscale	10.36 (7.43)	11.21 (9.41)	0.621^a^
DASS – stress subscale	20.12 (8.60)	22.17 (10.11)	0.283^a^
Binge Eating Scale	29.84 (8.11)	28.67 (7.33)	0.455^a^
Eating Disorder Examination Questionnaire	3.64 (1.10)	3.46 (0.95)	0.395^a^
	**N (%)**	**N (%)**	
DSM-5 diagnoses			0.597^b^
Binge eating disorder	30 (60.00)	36 (75.00)	
Bulimia nervosa	8 (16.00)	5 (10.42)	
OSFED/UFED	12 (24.00)	7 (14.58)	

*HDL* High-Density Lipoprotein, *LDL* Low-Density Lipoprotein, *SF-12* 12-item Short Form Health Survey, *DASS* Depression, Anxiety and Stress Scale, *OSFED* other specified feeding or eating disorder, *UFED* unspecified feeding or eating disorder. Note: All serum measurements were made in the fasted state

^a^ T test; ^b^ Fisher’s test

### Outcomes

No statistical differences between participants in either treatment group were found at assessment, nor for the interaction between treatment group and time for all outcomes examined in this study (see *p* values in Table 2 and Table 3 below). In other words, there was no significant difference between the two treatments (HAPIFED and CBT-E) with respect to any of the outcomes in this study, nor there were any significant differences between the two treatments in the pattern of change in outcomes over time.

**Table 2:** Biochemical and metabolic outcomes using imputation and mixed effects models – HAPIFED (*n* = 50) and CBT- E (*n* = 48)

	Group	Baseline		End of Treatment		Group	Time	Group x time
		Mean ± SE	Cohen’s d (95% CI)	Mean ± SE	Cohen’s d (95% CI)	***Chi sq, df*** ***(p value)***	***Chi sq, df*** ***(p value)***	***Chi sq, df*** ***(p value)***
Serum glucose, mg/dL	HAPIFED	93.51 ± 1.45	0.10 (−0.30 to 0.49)	92.50 ± 1.64	0.15 (−0.25 to 0.55)	0.22, 1 (0.636)	0.02, 1 (0.896)	0.14, 1 (0.713)
	CBT-E	94.49 ± 1.48		94.28 ± 1.74				
Serum insulin, ulU/mL	HAPIFED	13.57 ± 1.17	−0.11 (−0.51 to 0.29)	12.66 ± 1.29	−0.05 (−0.45 to 0.34)	0.30, 1 (0.581)	0.19, 1 (0.662)	0.09, 1 (0.769)
	CBT-E	12.64 ± 1.20		12.19 ± 1.32				
Serum triglycerides, mg/dL	HAPIFED	147.66 ± 17.78	−0.19 (−0.58 to 0.21)	145.39 ± 20.08	−0.18 (−0.58 to 0.22)	0.87, 1 (0.352)	0.11, 1 (0.735)	0.03, 1 (0.860)
	CBT-E	123.98 ± 18.17		119.03 ± 21.44				
Serum HDL cholesterol, mg/dL	HAPIFED	54.55 ± 1.52^A^	−0.07 (−0.46 to 0.33)	51.88 ± 1.85^B^	−0.13 (−0.52 to 0.27)	0.11, 1 (0.744)	4.14, 1 **(0.042)**	0.11, 1 (0.743)
	CBT-E	53.84 ± 1.55^A^		50.29 ± 1.72^B^				
Serum LDL cholesterol, mg/dL	HAPIFED	117.50 ± 4.20	−0.12 (−0.52 to 0.27)	109.75 ± 5.27	0.01 (−0.38 to 0.41)	0.38, 1 (0.540)	0.54, 1 (0.463)	0.30, 1 (0.585)
	CBT-E	113.84 ± 4.24		110.27 ± 4.92				

Normal reference value for adults (fasted state): serum glucose 60–100 mg/dL; serum basal insulin 1.9–23.0 ulU/mL; serum triglycerides <150 mg/dL; serum HDL cholesterol >40 mg/dL; serum LDL cholesterol 100–129 mg/dL

**Table 3:** Comparative metabolic, psychological and eating behaviour outcomes in the HAPIFED (*n* = 50) and CBT-E (*n* = 48) groups using imputation in all analyses and mixed effects models

	Group	Baseline		Middle of Treatment		End of Treatment		6-month follow-up		12-month follow-up		Group	Time	Group x time
		Mean ± SE	Cohen’s d (95% CI)	Mean ± SE	Cohen’s d (95% CI)	Mean ± SE	Cohen’s d (95% CI)	Mean ± SE	Cohen’s d (95% CI)	Mean ± SE	Cohen’s d (95% CI)	***Chi sq, df*** ***(p value)***	***Chi s, df*** ***(p value)***	***Chi sq, df*** ***(p value)***
Waist circumference (cm)	HAPIFED	105.18±1.52	−0.03 (−0.42 to 0.37)	n.a.	n.a.	104.13 ± 1.57	0.03 (−0.37 to 0.43)	103.95 ± 1.77	0.01 (−0.39 to 0.41)	104.04 ± 2.30	−0.04 (−0.44 to 0.36)	0.02, 1 (0.900)	1.36, 3 (0.714)	0.37, 3 (0.947)
	CBT-E	104.90±1.55		n.a.		104.48 ± 1.65		104.08 ± 1.71		103.45 ± 1.82				
Hip circumference (cm)	HAPIFED	118.83±1.13	0.00 (−0.39 to 0.40)	n.a.	n.a.	117.94 ± 1.19	−0.06 (−0.46 to 0.34)	116.93 ± 1.30	0.05 (−0.34 to 0.45)	116.73 ± 1.62	0.00 (−0.40 to 0.39)	0.00, 1 (0.991)	5.04, 3 (0.169)	0.70, 3 (0.874)
	CBT-E	118.84±1.15		n.a.		117.43 ± 1.26		117.41 ± 1.25		116.69 ± 1.35				
SF12 physical health component summary	HAPIFED	47.22±1.42	0.27 (−0.12 to 0.67)	47.83 ± 1.49	0.24 (−0.16 to 0.63)	49.42 ± 1.61	0.11 (−0.29 to 0.50)	50.70 ± 1.70	−0.04 (−0.44 to 0.36)	48.19 ± 1.84	0.06 (−0.34 to 0.45)	1.84, 1 (0.175)	1.25, 4 (0.870)	3.02, 4 (0.554)
	CBT-E	49.97±1.45		50.47 ± 1.69		50.64 ± 1.60		50.22 ± 1.78		48.93 ± 1.99				
SF12 mental health component summary	HAPIFED	34.31±1.78^B^	0.21 (−0.19 to 0.60)	37.60 ± 1.94^B^	0.07 (−0.32 to 0.47)	42.63 ± 2.21^A^	−0.01 (−0.41 to 0.39)	42.57 ± 2.06^A^	−0.08 (−0.48 to 0.32)	46.17 ± 2.58^A^	−0.07 (−0.47 to 0.32)	1.06, 1 (0.304)	10.56, 4 **(0.032)**	1.97, 4 (0.741)
	CBT-E	36.92±1.82^B^		38.63 ± 2.16^B^		42.46 ± 2.32^A^		41.23 ± 2.72^A^		44.80 ± 2.69^A^				
DASS-depression	HAPIFED	16.38±1.50	−0.01 (−0.41 to 0.38)	15.88 ± 1.64	0.00 (−0.39 to 0.40)	11.99 ± 1.64	0.02 (−0.37 to 0.42)	11.23 ± 2.02	0.07 (−0.33 to 0.47)	9.89 ± 1.93	0.13 (−0.27 to 0.53)	0.00, 1 (0.952)	5.06, 4 (0.281)	0.38, 4 (0.984)
	CBT-E	16.25±1.53		15.92 ± 1.77		12.26 ± 1.79		12.20 ± 1.91		11.78 ± 2.25				
DASS-anxiety	HAPIFED	10.36±1.32	0.09 (−0.31 to 0.49)	11.10 ± 1.60	−0.04 (−0.44 to 0.35)	8.49 ± 1.47	−0.05 (−0.45 to 0.35)	8.34 ±1.92	0.11 (−0.29 to 0.51)	7.19 ± 2.85	0.11 (−0.28 to 0.51)	0.20, 1 (0.654)	1.65, 4 (0.800)	2.15, 4 (0.708)
	CBT-E	11.21±1.35		10.57 ± 1.88		7.90 ± 1.85		10.07 ± 2.56		9.54 ± 3.14				
DASS-stress	HAPIFED	20.12±1.29^A^	0.22 (−0.17 to 0.62)	18.83 ± 1.45^A^	0.11 (−0.29 to 0.51)	15.00 ± 1.34^B^	0.00 (−0.40 to 0.40)	15.11 ± 1.90^B^	0.09 (−0.31 to 0.48)	12.92 ± 1.86^B^	0.09 (−0.31 to 0.47)	1.23, 1 (0.268)	25.88, 1 **(< .0001)**	0.66, 4 (0.956)
	CBT-E	22.17±1.32^A^		20.03 ± 1.66^A^		14.99 ± 1.56^B^		16.17 ± 1.64^B^		14.02 ± 1.83^B^				
Binge Eating Scale	HAPIFED	29.84±1.19^A^	−0.14 (−0.54 to 0.26)	20.41 ± 1.38^A^	−0.11 (−0.50 to 0.29)	15.19 ± 1.36^B^	−0.15 (−0.55 to 0.24)	13.94 ± 1.42^B^	0.06 (−0.34 to 0.45)	11.30 ± 1.64^B^	0.08 (−0.32 to 0.48)	0.47, 1 (0.492)	104.23, 4 **(< 0.001)**	2.19, 4 (0.700)
	CBT-E	28.67±1.22^A^		19.36 ± 1.43^A^		13.61 ± 1.57^B^		14.50 ± 1.46^B^		12.21 ± 1.59^B^				
Eating Disorder Examination QuestionnaireGlobal score	HAPIFED	3.64±0.17	−0.15 (−0.54 to 0.25)	2.84 ±0.19	−0.14 (−0.53 to 0.26)	2.21 ± 0.19	−0.17 (−0.57 to 0.22)	2.11 ±0.19	−0.05 (−0.45 to 0.34)	1.94 ± 0.24	−0.01 (−0.41 to 0.39)	0.54, 1 (0.463)	84.94, 4 **(< 0.001)**	0.90, 4 (0.924)
	CBT-E	3.46±0.17		2.66 ±0.19		1.97 ± 0.21		2.04 ±0.20		1.92 ± 0.21				

*CBT-E* cognitive behavioural therapy-enhanced, *HAPIFED* Healthy APproach to weIght management and Food in Eating Disorders, *SF-12* 12-item short form health survey

(^A^) and (^B^) present different means, according to contrasts with Bonferroni correction

#### Indicators of metabolic health: blood markers and waist and hip circumferences

Table 2 shows the data for the five fasting blood tests (serum glucose, serum insulin, serum triglycerides and serum HDL and LDL), analysed at two timepoints (baseline and end of treatment). There were decreases over time to the end of treatment but these were not significant, with the exception of a reduction in serum HDL levels (*p* = 0.042). There were no significant differences between intervention groups in the time effect analyses. Of note, participants’ serum parameters were within the normal range [42–44] at baseline, and remained so until the end of treatment.

Participant’s waist and hip circumferences were measured at four timepoints (baseline, end of treatment, 6-month and 12-month follow-ups). There were no statistical or clinically significant differences in waist or hip circumference measurements over time. There was no statistically significant difference between-group comparisons in waist (*p* = 0.947) and hip (*p* = 0.874) measures (see Table 3).

#### Physical and mental health-related quality of life (SF-12)

At baseline, participants’ Physical Component Summary SF12 scores were close to a healthy level of 50 in both groups (Table 1), and scores did not change significantly over time (baseline to the 12-month follow-up) (*p* = 0.870). For the Mental Component Summary SF12 measure, a significant time effect improvement was found (*p* = 0.032) at the end of treatment and this continued until the 12-month follow-up (see Table 3).

#### General psychiatric symptoms – depression, anxiety and stress (DASS-21)

By the 12-month follow-up, all individuals in the HAPIFED group had reduced depression, anxiety and stress scores to within the normal levels, whereas those in the CBT-E group had reduced scores to within a mild level for depression and anxiety, and to within the normal level for stress. For DASS-depression (*p* = 0.281) and DASS-anxiety (*p* = 0.800), no significant time effects were found. However, statistical significance was found in the time effect for both groups for the reduction of stress from the end of treatment onwards (*p* < 0.001), as shown in Table 3.

#### Severity of binge eating on the BES and global self-reported ED symptoms on the EDE-Q

These were evaluated at five time-points. For both severity of binge eating and global self-reported ED symptoms, a significant time effect was found (*p* < 0.001) at the end of treatment and at the 12-month follow-up. Participants in both groups started the intervention at a severe level of the BES (> 27 points), and finished the follow-up (i.e., the 12-month follow-up) at a normal level (< 17 points) (see Table 3).

#### Remission of DSM-5 criteria for ED diagnoses

A secondary analysis compared the proportions of those who no longer met DSM-5 criteria for BED, BN or OSFED/UFED at three different time points: the end of treatment [61% (*n* = 22) in HAPIFED versus 67% (*n* = 20) in CBT-E]; at 6-month follow-up [68% (*n* = 19) in HAPIFED versus 77% (*n* = 17) in CBT-E]; and at 12-month follow-up [70% (*n* = 21) in HAPIFED versus 45% (*n* = 13) in CBT-E]. These data favored the HAPIFED arm at 12-month follow up (*p* = 0.050), however when applying imputated data analyses, these differences did not reach statistical significance.

## Discussion

To our knowledge, this is the first RCT to test an integrated CBT-E with a behavioural weight loss psychological intervention, with no pharmacological support, for people with recurrent binge eating episodes comorbid with a high BMI. A previous publication presented the results of the primary outcome (weight loss) with similar results for HAPIFED and CBT-E, and in both interventions, there was no statistically or clinically significant weight loss from baseline, and no difference between groups [15]. For the secondary outcomes of the trial, as reported in this study, no statistical significance was found for differences between the interventions, but both HAPIFED and CBT-E showed significant time effect improvements in stress and ED symptoms from baseline, that were apparent from the end of treatment onwards. This manuscript adds to the former publication [15] in providing information on metabolic parameters, other general and eating disorder psychopathology, and diagnostic status outcomes.

It is known that individuals with BN or BED can present impaired hematologic and metabolic profiles [45, 46]. This study comprised people with a high BMI and waist circumferences above the normal range (> 102 cm for men and > 88 cm for women), according to the National Cholesterol Education Program of Brazil (2002) [47]. However, blood tests for participant biomarkers were in the normal range at baseline, with small improvements over time. This was thus a sample of “metabolically healthy” people with mean BMI (33.68 kg/m2) in the range of Class 1 obesity. This may explain why there was little change in physical health status that was already close to or within normal parameters.

In contrast, the mental health-related quality of life improved over the 12-month follow-up. While there were nonsignificant reductions in general psychopathology (i.e. depression, anxiety), there were improvements in stress levels. However, there was no significant weight loss. In the present study, ED symptoms also improved significantly in the 12-month follow-up (*p* < 0.001). These results are in line with data presented by a systematic review that evaluated psychological and ED outcomes (among others) in studies that aimed to test the effects of weight loss interventions in individuals with high BMI where positive ED outcomes were described, e.g., reduction of binge eating frequency [48]. The authors highlighted that ED symptoms, health-related quality of life and weight status are associated, and added that, although the interventions that combine psychological or behavioural elements are less effective in weight loss, they can favour improvements in ED and general psychopathology. They suggest a dual focus for weight loss interventions including weight loss strategies (lifestyle approaches, pharmacotherapy or surgery) and psychological well-being (evaluation and treatment of disordered eating symptoms) [48]. Pataky et al. (2018) [49] showed that psychological parameters (e.g., eating, shape and weight concerns, disinhibition and self-esteem) can be improved in groups of participants that lost weight as well in groups that remained weight stable after 12 months of a multidisciplinary and lifestyle weight loss program. They hypothesized that the stabilization of weight can help the prevention of further weight gain during one year [49]. It may be that weight stability can be as important as weight loss in some lifestyle respects for individuals with a high BMI receiving treatment.

Further, it may have been expected that CBT-E would be superior concerning eating disorder pathology as the treatment dose is higher towards eating disorder pathology (as no sessions are needed to address weight). In the present trial there were more than adequate sessions to deliver CBT-E (usually delivered over 20 sessions and here over 30 sessions) and HAPIFED could be conceptualized as a CBT-E ‘plus’ therapy – the ‘plus’ begin to address people’s concerns overtly about weight loss and provide more detailed nutritional and activity advice, albeit that two of the three additional modules were not administered in HAPIFED. Finally, a meta-analysis has found mixed results for CBT’s superiority to other psychological therapies for treatment across a range of eating disorders [50], and further studies are needed to assess CBT against other active psychological interventions.

This study has several strengths and limitations. Strengths of this study include the randomized design that introduced a new and original integrative intervention that focused on a transdiagnostic approach for individuals with recurrent binge eating episodes diagnosed with BN, BED, and OSFED/UFED. HAPIFED addressed both ED psychopathology and weight management, stabilizing body weight and improving ED and general psychopathology and health-related quality of life, albeit to no greater extent than CBT-E. This is important as studies that compare psychological treatments for individuals with BED and a high BMI usually do not examine the impact of interventions on metabolic and physical health measures beyond weight. Another strength of this study is that all data and an intention to treat approach were used in the statistical analysis and the participants as well the statistician were blinded to treatment allocation, decreasing risk of bias. Limitations of this study were that this study evaluated only women who were Caucasian and generally well educated, there was only a small number of participants with BN, and there was moderate attrition from the trial, limiting generalizability of the data.

## Conclusions

This analysis of secondary outcomes found that participants’ improved psychological health did not differ between HAPIFED and CBT-E, and there was little change in physical health outcomes with either treatment. It is likely that more intense weight loss interventions, possibly in populations with greater levels of BMI and metabolic dysregulation, would need to be investigated in order to see benefits of HAPIFED over CBT-E for weight, metabolic and physical health outcomes. However, care would be needed to ensure that any greater intensity of the weight loss component of HAPIFED does not interfere with the psychological benefits of the treatment. These are complex conditions involving mental and physical/medical disorders, and further studies are needed examining effects of combined psychological and other interventions (e.g. pharmacotherapy) in individuals with recurrent binge eating episodes with high BMI.

## Supplementary Information

**Additional file 1: Supplementary File 1.** Changes from the published protocol paper (Palavras et al., Trials 2015 16:578) and as reported in the Trial registration of the present paper.

**Additional file 2: Supplementary File 2.** Description of the topics of each session for the HAPIFED and CBT-E as implemented in the present study.

**Additional file 3: Supplementary File 3.** Summary of similarities and differences between HAPIFED and Cognitive Behaviour Therapy–Enhanced therapies as implemented in the present study.

## Data Availability

De-identified data are available for collaborative projects upon request and with appropriate Ethics approval. Requests for access to the data should be made to the corresponding author Phillipa Hay (p.hay@westernsydney.edu.au).

## Abbreviations

BED: Binge Eating Disorder
BES: Binge Eating Scale
BMI: Body Mass Index
BN: Bulimia Nervosa
BWLT: Behavioural Weight Loss Therapy
CBT: Cognitive Behavioural Therapy
CBT-E: Cognitive Behavioural Therapy – Enhanced
DALYs: Disability Adjusted Life Years
DASS-21: Depression Anxiety and Stress Scale – Short Form 21 Items
DSM-5: Diagnostic and Statistical Manual of Mental Disorders, fifth edition
ED: Eating Disorder
EDs: Eating Disorders
EDE: Eating Disorder Examination
EDE-Q: Eating Disorder Examination Questionnaire
GEE: Generalized Estimation Equation
HAPIFED: Health Approach to Weight Management and Food in Eating Disorder
HDL: High Density Lipoprotein
ICD-11: International Classification of Diseases, eleventh edition
ITT: Intention to Treat
LDL: Low Density Lipoprotein
MCMC: Marko Chain Monte Carlo
MINI: Mini International Neuropsychiatric Interview
OSFED: Other Specified Feeding or Eating Disorder
PROATA: Núcleo de Atenção aos Transtornos Alimentares
RCT: Randomized Controlled Trial
SF-12: The 12-item Short Form Health Survey
UFED: Unspecified Feeding or Eating Disorder
UNIFESP: Universidade Federal de São Paulo
