# Supplementary material for: Physical and mental health outcomes of an integrated cognitive behavioural and weight management therapy for people with an eating disorder characterized by binge eating and a high body mass index: a randomized controlled trial
Source: BMC Psychiatry. 2022 May 24;22:355. doi: 10.1186/s12888-022-04005-y (PMC9131673; doi:10.1186/s12888-022-04005-y)
Supplement: Supplementary file 1 — Additional file 1: Supplementary File 1. Changes from the published protocol paper (Palavras et al., Trials 2015 16:578) and as reported in the Trial registration of the present paper. [file 12888_2022_4005_MOESM1_ESM.docx]

**Supplementary File 1 - Changes from the published protocol paper (Palavras et al., Trials 2015 16:578) and as reported in the Trial registration of the present paper**

1. Subthreshold eating disorder categories – Other Specified Feeding or Eating Disorder (OSFED) i.e., Bulimia Nervosa and Binge Eating Disorder of low frequency and/or limited duration and Unspecified Feeding or Eating Disorder (UFED) were added to the inclusion criteria.

We planned to include a primary eating disorder diagnosis of BN or BED type according to the Diagnostic and Statistical Manual of Mental Disorders, fifth edition (DSM-5) and/or the proposed International Classification of Diseases, eleventh version (ICD-11) criteria. The ICD-11 criteria are broader and so include people who with DSM would have OSFED or UFED. We also had people who were otherwise eligible, but who had OSFED or UFED or OFED (ICD-11) and wanted to include them as their symptoms and quality of life impairment were similar – we subsequently have published the results of this analysis in Palavras MA, Hay P, and Claudino A. An investigation of the Clinical Utility of the Proposed ICD-11 and DSM-5 Diagnostic Schemes for Eating Disorders Characterized by Recurrent Binge Eating in People with a High BMI. Nutrients 2018 Nov 13;10(11):1751. doi: 10.3390/nu10111751.

02. The assessment of a middle of treatment was not mentioned in the protocol paper (Palavras et al., Trials, 2015), but was included in the registration of this trial in clinicaltrials.gov, as well as in the paper presenting the primary outcomes (Palavras et al., 2021) and this current manuscript.

03. The evaluation of blindness integrity was not mentioned in the Trial registration, but was inserted in the protocol paper and evaluated in the above-mentioned paper presenting the primary outcomes (Palavras et al., 2021).

04. Protocol violation: Seven participants received an antidepressant prescription due to worsening of depressive symptoms during the intervention. The use of antidepressant medication for people with Bulimia Nervosa or Binge Eating Disorder can result in weight gain. These participants were retained in the trial and the analyses, instead of being excluded from the analyses, as the senior team of supervisors was advised by the treating clinician that these participants would benefit from continuing to receive the psychological treatment.
