# Supplementary material for: Physical and mental health outcomes of an integrated cognitive behavioural and weight management therapy for people with an eating disorder characterized by binge eating and a high body mass index: a randomized controlled trial
Source: BMC Psychiatry. 2022 May 24;22:355. doi: 10.1186/s12888-022-04005-y (PMC9131673; doi:10.1186/s12888-022-04005-y)
Supplement: Supplementary file 2 — Additional file 2: Supplementary File 2. Description of the topics of each session for the HAPIFED and CBT-E as implemented in the present study. [file 12888_2022_4005_MOESM2_ESM.docx]

Supplementary File 2: Description of the topics of each session for the HAPIFED and CBT-E as implemented in the present study.

|  | **HAPIFED** | **CBT-E** |
| --- | --- | --- |
| **Group Session** | **Topics** | **Topics** |
| 01 | Establishing real-time self-monitoring and education about weight, weight goals and weight change in treatment | Establishing real-time self-monitoring and education about weight, weight goals and weight change in treatment |
| 02 | Psychoeducation about eating problems | Psychoeducation about eating problems |
| 03 | Famine reaction/ Why the diets fail? | Establishing regular eating |
| 04 | Establishing regular eating | Addressing purging |
| 05 | Psychoeducation about healthy and unhealthy exercise | Addressing feelings of fullness |
| 06 | Addressing purging | Addressing excessive exercise |
| 07 | Addressing the healthy eating for weight loss | Involving significant others |
| 08 | Addressing feelings of fullness | Conducting a joint review of progress/Identifying barriers to change |
| 09 | Addressing excessive exercise | Conducting a joint review of progress/Identifying barriers to change |
| 10 | Involving significant others | Identifying over-evaluation and its consequence |
| 11 | Conducting a joint review of progress/Identifying barriers to change | Enhancing the importance of other domains for self-evaluation |
| 12 | Strategies for relaxation | Addressing shape checking and avoidance |
| 13 | Liking your body/Identifying over-evaluation and its consequence | Continuation of session 12 |
| 14 | Enhancing the importance of other domains for self-evaluation | Addressing “feeling fat” |
| 15 | Self-nurturing activities | Exploring the origins of the over-evaluation |
| 16 | Addressing shape checking and avoidance | Learning to control the ED mindset |
| 17 | Continuation of session 16 | Addressing dietary restraint and over-evaluation of control over eating |
| 18 | Addressing “feeling fat” | Continuation of session 17 |
| 19 | Exploring the origins of the over-evaluation | Addressing event-related changes in eating |
| 20 | Continuation of session 19 | Addressing mood-related changes in eating |
| 21 | Addressing life skills, interpersonal functioning and relationships | Addressing clinical perfectionism |
| 22 | Learning to control the ED mindset | Continuation of session 21 |
| 23 | Addressing dietary restraint and over-evaluation of control over eating | Addressing core low self-esteem |
| 24 | Continuation of session 23 | Continuation of session 23 |
| 25 | Addressing event-related changes in eating | Addressing interpersonal problems |
| 26 | Addressing mood-related changes in eating | Continuation of session 25 |
| 27 | Addressing the end of treatment | Addressing the end of treatment |
| 28 | Addressing the end of treatment | Addressing the end of treatment |
| 29 | Addressing the end of treatment | Addressing the end of treatment |

HAPIFED = Healthy APproach to WeIght management and Food in Eating Disorders

CBT-E – Cognitive Behavioural Therapy-Enhanced
