# Supplementary material for: Physical and mental health outcomes of an integrated cognitive behavioural and weight management therapy for people with an eating disorder characterized by binge eating and a high body mass index: a randomized controlled trial
Source: BMC Psychiatry. 2022 May 24;22:355. doi: 10.1186/s12888-022-04005-y (PMC9131673; doi:10.1186/s12888-022-04005-y)
Supplement: Supplementary file 3 — Additional file 3: Supplementary File 3. Summary of similarities and differences between HAPIFED and Cognitive Behaviour Therapy–Enhanced therapies as implemented in the present study. [file 12888_2022_4005_MOESM3_ESM.docx]

Supplementary File 3: Summary of similarities and differences between HAPIFED and Cognitive Behaviour Therapy –Enhanced therapies as implemented in the present study.

| **Included** | **HAPIFED** | **CBT-E** |
| --- | --- | --- |
| **Use of Eating Disorder (ED) CBT formulation** | Yes, includes high BMI | Yes, includes ED only |
| **Psychoeducation** | Yes, ED and high BMI | Yes, ED only |
| **Nutritional counselling** | Yes, dietician led | Yes, not dietician led |
| **Behavioural monitoring** | Yes, with appetite cues | Yes |
| **Multidisciplinary** | Yes | No |
| **Session / duration** | 30 sessions/6 months | 30 sessions/6 months |
| **Weight loss management** | Yes | No |
| **Behavioural activation** | Yes | No |
| **Healthy exercise encouraged** | Yes | No |
| **Emotion regulation skills** | Yes | Yes |

ED = Eating Disorders

HAPIFED = Healthy APproach to WeIght management and Food in Eating Disorders

BMI = Body mass Index (kg/m^2^)

**Adapted from** Palavras MA, Hay P, Touyz S, Sainsbury A, da Luz F, Swinbourne J, Estella NM, Claudino A. Comparing cognitive behavioural therapy for eating disorders integrated with behavioural weight loss therapy to cognitive behavioural therapy-enhanced alone in overweight or obese people with bulimia nervosa or binge eating disorder: study protocol for a randomised controlled trial. Trials. 2015 Dec;16(1):1-0.. Used with author permission under Creative Commons Licence conditions.
